# Blood culture surveillance in a secondary care hospital in Benin: epidemiology of bloodstream infection pathogens and antimicrobial resistance

**DOI:** 10.1186/s12879-022-07077-z

**Published:** 2022-02-03

**Authors:** Sien Ombelet, Gutemberg Kpossou, Carine Kotchare, Esenam Agbobli, Frédéric Sogbo, Faridath Massou, Katrien Lagrou, Barbara Barbé, Dissou Affolabi, Jan Jacobs

**Affiliations:** 1 Institute of Tropical Medicine, Antwerp, Belgium; 2 Department of Microbiology, Immunology & Transplantation, KU Leuven, Leuven, Belgium; 3 Hôpital Saint Jean de Dieu de Boko, Boko, Benin; 4 Centre National Hospitalier Universitaire Hubert Koutougou MAGA de Cotonou (CNHU-HKM), Cotonou, Benin; 5 National Reference Laboratory for Mycobacteria, Cotonou, Benin; 6 Clinical Department of Laboratory Medicine, University Hospitals Leuven, Leuven, Belgium

**Keywords:** Sepsis, Bloodstream infections, Antimicrobial resistance, Low- and middle-income countries, sub-Saharan Africa

## Abstract

**Background:**

Although global surveillance of antimicrobial resistance (AMR) is considered key in the containment of AMR, data from low- and middle-income countries, especially from sub-Saharan Africa, are scarce. This study describes epidemiology of bloodstream infections and antimicrobial resistance rates in a secondary care hospital in Benin.

**Methods:**

Blood cultures were sampled, according to predefined indications, in BacT/ALERT FA Plus and PF Plus (bioMérieux, Marcy-l’Etoile, France) blood culture bottles (BCB) in a district hospital (Boko hospital) and to a lesser extent in the University hospital of Parakou. These BCB were incubated for 7 days in a standard incubator and twice daily inspected for visual signs of growth. Isolates retrieved from the BCB were processed locally and later shipped to Belgium for reference identification [matrix-assisted laser desorption/ionization time-of-flight spectrometry (MALDI-TOF)] and antibiotic susceptibility testing (disk diffusion and E-tests).

**Results:**

From October 2017 to February 2020, 3353 BCB were sampled, corresponding to 3140 blood cultures (212 cultures consisting of  > 1 BCB) and 3082 suspected bloodstream infection (BSI) episodes. Most of these cultures (n = 2471; 78.7%) were sampled in children < 15 years of age. Pathogens were recovered from 383 (12.4%) cultures, corresponding to 381 confirmed BSI. 340 of these pathogens were available and confirmed by reference identification. The most common pathogens were *Klebsiella pneumoniae* (n = 53; 15.6%), *Salmonella* Typhi (n = 52; 15.3%) and *Staphylococcus aureus* (n = 46; 13.5%). AMR rates were high among *Enterobacterales*, with resistance to third-generation cephalosporins in 77.6% of *K. pneumoniae* isolates (n = 58), 12.8% of *Escherichia coli* isolates (n = 49) and 70.5% of *Enterobacter cloacae* isolates (n = 44). Carbapenemase production was detected in 2 *Escherichia coli* and 2 *Enterobacter cloacae* isolates, all of which were of the New Delhi metallo-beta lactamase type. Methicillin resistance was present in 22.4% of *S. aureus* isolates (n = 49).

**Conclusion:**

Blood cultures were successfully implemented in a district hospital in Benin, especially among the pediatric patient population. Unexpectedly high rates of AMR among Gram-negative bacteria against commonly used antibiotics were found, demonstrating the clinical and scientific importance of clinical bacteriology laboratories at this level of care.

**Supplementary Information:**

The online version contains supplementary material available at 10.1186/s12879-022-07077-z.

## Background

Access to clinical bacteriology services in low- and middle-income countries (LMIC) is increasingly recognized as a crucial component in the adequate management of severe bacterial infections and in the prevention of antimicrobial resistance (AMR) [1–4]. LMIC are disproportionally affected by AMR and hospital-acquired infections [5], but laboratories in these countries are often ill-equipped to offer diagnosis of resistant infections in anything other than tertiary centres [2, 6]. Especially sub-Saharan Africa is still underserved in terms of quality-assured clinical bacteriology services [6]. As a consequence, meta-analyses describing antimicrobial resistance rates in Africa describe high rates of resistance but emphasize the scarcity of data, the bias towards tertiary centres in urban areas and the lack of microbiological quality control in most studies [7, 8]. The financial, logistic and infrastructural challenges associated with clinical bacteriology in LMIC have been described elsewhere [2, 9–12]. However, it has also been demonstrated that acceptable quality bacteriology can be achieved in these settings when provided with adequate financial, logistic and supervisory support [3]. Availability of bacteriology testing is also key to implementation of antimicrobial stewardship programs in LMIC [13, 14]. Antimicrobial stewardship, defined as the effort to promote appropriate use of antimicrobials to improve patient outcomes, reduce antibiotic resistance and decrease spread of multi-drug resistance [15], relies on knowledge of local AMR epidemiology to effectively guide clinicians towards appropriate treatments.

**Fig. 1:**
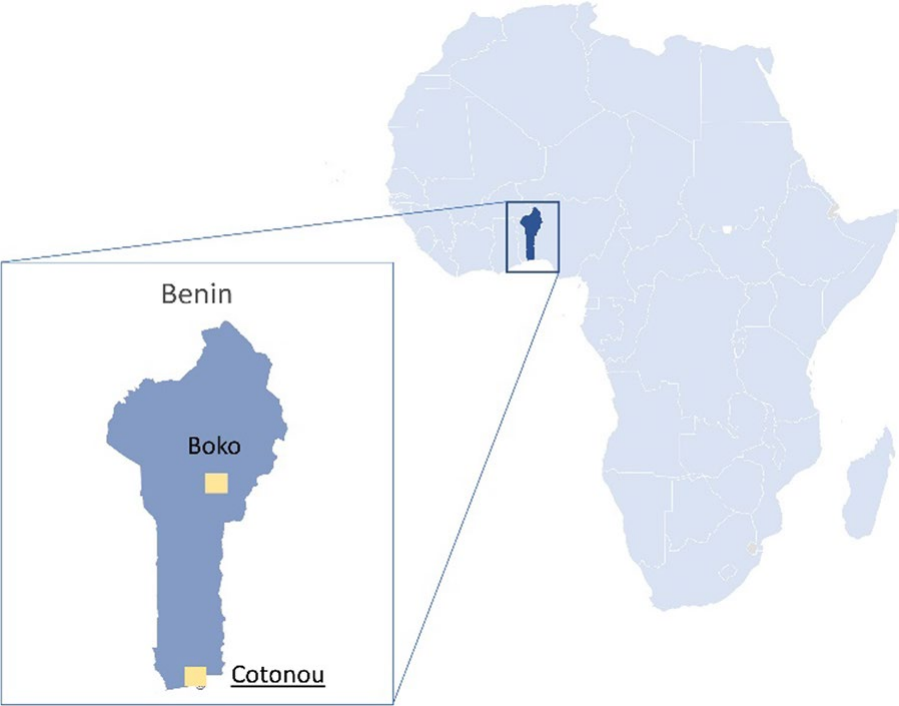
Location of Benin within Western Africa and location of Boko within Benin. Boko is situated in the Borgou province, close to the second largest city of the country, Parakou. The capital city, Cotonou, is located at 400 km distance. (Map generated with “Maps” function in Excel, Microsoft. © GeoNames, Microsoft, Tom Tom, Wikipedia)

No antimicrobial resistance data of clinical isolates have been reported so far from Benin. Benin is a West-African country which was recently upgraded from low-income to lower-middle-income status according to the World Bank [16] (Fig. 1). A blood culture implementation project, with embedment in clinical care, was started in a semi-rural hospital in Benin, Boko Hospital. The primary objective of this project was to report antimicrobial resistance rates and epidemiology of bloodstream infections from a semi-rural hospital in Benin. The secondary objective was to showcase an example of blood culture implementation in such a setting, including monitoring of blood culture quality indicators such as contamination rate and volume of blood sampled. This project was carried out by the Centre National Hospitalier Universitaire Hubert Koutougou Maga de Cotonou (CNHU-HKM, Benin) in collaboration with the Institute of Tropical Medicine of Antwerp (Belgium) and the National Reference Laboratory for Mycobacteria of Benin, it was logistically supported by the non-governmental organization LUMOS/Memisa.

## Methods

### Study site

Hôpital de Saint Jean de Dieu de Boko (further referred to as Boko hospital) is a 95-bed district hospital in the department of Borgou, Benin. It serves a population of 423,530 citizens in the health zone Parakou-N’dali, one of four health zones in Borgou. It offers dedicated services of paediatrics, internal medicine, surgery, maternity, radiology, physiotherapy and emergency care. As Boko Hospital became known locally for performing blood cultures as part of this study, blood cultures were occasionally submitted by the Centre Hospitalier Universitaire Departemental (CHUD) of Parakou. Parakou is the nearest city to Boko, and the second largest in the country, with a population of around 206,667 citizens.

### Study design and set-up

A prospective antimicrobial resistance surveillance study on blood culture isolates was initiated in September 2017 in Boko hospital. The data presently described are surveillance data from October 2017 to February 2020. At the start of the surveillance project, the importance of blood cultures and sampling indications were discussed with laboratory and medical staff of Boko hospital. Regular feedback (three times per year) was given to the medical and nursing staff on evolutions in blood culture sampling, contamination of blood cultures, epidemiology of bloodstream pathogens and antimicrobial resistance. No such feedback and follow-up were provided to CHUD Parakou.

### Blood culture sampling in Boko hospital

Sampling indications are presented in Box 1 and were advertised in each ward (in French). The blood culture request form, including sampling indications, is available as Additional file 1: File 3. As consumables were provided by the capacity building project, the cost of blood culture could be minimized to preclude barriers to access. Practical and theoretical trainings in blood culture sampling were offered to nursing and laboratory staff yearly (September 2017, September 2018 and October 2019). BacT/ALERT FA Plus and PF Plus (bioMérieux, Marcy-l’Etoile, France) blood culture bottles (BCB) were used. For adults, two aerobic BCB were sampled. For children, 1 pediatric BCB was sampled. Volume recommendations for sampling were part of the training and are presented in Box 2. Antisepsis of the skin before sampling was performed with a two-step procedure: in a first step, the skin was cleaned with ethanol 70%; this step was repeated until swabs were clean. In the second step, povidone iodine was applied to the puncture site. In October 2019, the second step antiseptic was switched to chlorhexidine 2% in isopropyl alcohol 70% due to persistent problems with blood culture contamination.

**Box 1:** Indications for blood culture sampling as advertised in the wards [4]

1. Fever (axillary T° ≥ 38 °C) OR hypothermia (axillary T° ≤ 36 °C)
2. AND one of the following signs of severity
A. Hypotension (systolic blood pressure ≤ 100 mmHg)
B. Confusion
C. Increased respiratory rate (≥ 22/min)
D. Suspicion of severe localized infection
a. Pneumonia
b. Meningitis
c. Osteomyelitis
d. Complicated urinary tract infection
e. Abscess
f. Skin or soft tissue infection
g. Abdominal infection
E. Suspicion of other severe infection
a. Severe malaria
b. Typhoid fever
c. Other
F. Neonatal infection

**Box 2:** Recommendations for volume of blood culture sampling as instructed during blood sampling trainings [4]

Age	Volume of blood to be sampled
< 1 month of age	0.5–2 mL
1–36 months of age	1–4 mL
≥ 36 months of age	≥ 4 mL
≥ 15 years of age	8–12 mL

### On-site laboratory procedures

Upon receipt in the laboratory, BCB were incubated in a standard incubator (as opposed to a blood culture automate) and visually evaluated twice daily for signs of growth (details in Additional file 1: File 1 and 4). Upon signs of growth, a Gram stain was performed and a subculture was done. From September 2018 onwards, bottles were weighed upon reception at the laboratory with a Kern pocket balance (Kern & Sohn GmbH, Balingen, Germany) to determine sampled blood volume (see below). End of February 2019, a blind subculture (regardless of visual signs of growth) on day 1 of incubation (after one overnight incubation) of all BCB was implemented.

Local identification and antibiotic susceptibility testing (AST) of bacteria was done using conventional phenotypic testing [17, 18] and disk diffusion, respectively. Clinical & Laboratory Standards Institute (CLSI) breakpoints were used for interpretation of AST [19] (see Additional file 1 for details). A thick blood film (Giemsa stain) was assessed for presence of *Plasmodium* parasites when clinically suspected. These results were reported to clinicians as part of routine laboratory work-up.

Isolates of pathogens retrieved from blood culture were stored locally at − 80 °C (Microbank™, Pro Lab Diagnostics Inc, Ontario, Canada), inoculated on tryptic soy agar tubes before transport and shipped to the Institute of Tropical Medicine in Antwerp, Belgium.

### Reference laboratory work-up of bacterial isolates

Upon arrival in Belgium, the isolates were identified using matrix-assisted laser desorption/ionization time-of-flight mass spectrometry (MALDI-TOF MS), using a Microflex™ device (Bruker Daltonics, Massasuchetts, USA) with MALDI Biotyper® software (MBT 7854 MSP Library) at the University Hospitals Leuven (see Additional file 1: File 2 for details) Only MALDI-TOF confirmed isolates were taken into account in the analysis of key pathogens and antibiotic susceptibility testing.

All *Enterobacterales*, Gram-negative non-fermenting organisms (further referred to as non-fermenters) for which CLSI disk diffusion breakpoints were available, *Enterococcus* species and *Staphylococcus aureus* were tested for antibiotic susceptibility, using disk diffusion (Neo-Sensitabs) and E-test® (bioMérieux) when Minimal Inhibitory Concentration (MIC) was needed for interpretation (See Additional file 1: File 2 for details regarding reference isolate testing). CLSI breakpoints (M100-S30) were used for interpretation [20]. Extended spectrum beta-lactamase (ESBL) production was assessed for suspected Gram-negative isolates (showing ceftazidime diameters < 23 mm or ceftriaxone diameters < 26 mm) by performing double disk testing with cefotaxime 30 µg and ceftazidime 30 µg, both with and without clavulanic acid (Neo-Sensitabs), as described by CLSI [20]. Isolates suspected of carbapenemase production (meropenem diameter < 25 mm, or < 28 mm in combination with piperacillin-tazobactam and/or temocillin resistance) [21] were further assessed using meropenem E-test, combination disk testing (KPC+MBL detection kit, Rosco Diagnostica) and immunochromatographic lateral flow assays for detection of carbapenemase (RESIST-4 O.K.N.V., Coris Bioconcept, Gembloux, Belgium). These lateral flow tests detect KPC, OXA-48, NDM and VIM carbapenemase enzymes with high reliability [22–24]. Quality control of AST was performed daily (see Additional file 1: File 2 for details).

Isolates were de-duplicated (isolates from different cultures from the same episode were included only once if the antibiogram was the same). For cultures containing dimorphic isolates from the same species, antibiotic susceptibility was tested for both isolate morphotypes; when resulting antibiograms were different, both isolates were included in the analysis of AMR rates (but not in analysis of key pathogens).

### Volume of blood sampled

Volume of blood sampled was estimated by subtracting the mean empty weight of each new lot of BacT/ALERT FA and PF bottles (determined on ten bottles) from the weight of the filled bottle and dividing the result by the density of blood (= 1.06 g/mL).

### Data collection

Demographic and clinical data (such as administration of antibiotics < 24 h before sampling) were obtained from the blood culture request forms (template available as Additional file 1: File 3). Laboratory data were extracted from the laboratory worksheets (available as Additional file 1: File 4). These data were transferred locally to an Excel database (Microsoft, Washington, USA) by study collaborators SO, GK and CK. Data were anonymized after data cleaning. Indications for blood culture sampling, as filled in on the request forms, were converted to coded data on presumed focus of infection: generalized infection (when no localized infection was indicated on the form), abdominal infection, respiratory infection (i.e., suspicion of pneumonia or upper respiratory tract infections), central nervous system (i.e., suspicion of meningitis), urinary/genital (i.e., suspicion of complicated urinary tract infection or indication of genital infection) and purulent infections (i.e., suspicion of skin/soft tissue, osteomyelitis, abscess). Cleaned Excel databases were imported into R (R Foundation for Statistical Computing, Vienna, Austria).

### Data analysis

Data analysis was done using Excel (Microsoft Office 2019) and RStudio (R version 4.0.2). Univariable and multivariable logistic regression models were constructed in R to investigate associations between blood culture growth and clinical or demographic variables such as age, sex, prior antibiotic treatment, thick blood film (malaria microscopy) results, site of sampling (Boko or Parakou), presence of healthcare-associated infections and culture volume; similar models were constructed to look for associations between multidrug resistance and age, sex, prior antibiotic treatment or healthcare-associated infections. Age and sex were included as basic demographic variables. The other variables included in the models were identified as theoretically influencing odds of growth or odds of antimicrobial resistance, respectively. Thick blood film results and culture volume were not used for the final multivariable models, as there were too many missing data for these variables. Contamination proportions were compared using chi-square tests. Median age was compared using Wilcoxon rank sum tests. Statistical significance was defined as p-values < 0.05.

### Definitions

A blood culture was defined as any number of BCB (recommended: 2 for adults, 1 for children < 15 years) sampled at the same moment. Contamination was defined as isolation of any of the following bacterial species [17]: coagulase-negative *Staphylococcus* species, *Bacillus* species, *Corynebacterium* species, *Cutibacterium* species or *Micrococcus* species. These isolates were considered contaminants in all cases as the patient population in Benin had no indwelling central catheters or other foreign materials implanted. All other bacterial or fungal species isolated from blood culture were considered pathogens. Contamination rate was calculated as the number of contaminated bottles divided by the total number of bottles sampled (each bottle representing a separate venipuncture) [17]. A suspected bloodstream infection (BSI) episode was defined as all blood cultures sampled within a 14-day interval from the first culture. A culture-confirmed BSI episode was defined as either the initial recovery of a pathogen from a suspected episode, the recovery of a pathogen different from the initial pathogen ≥ 48 h after recovery of the first pathogen, or the recovery of the same pathogen after at least a 14-day interval [25]. Pathogen rate was defined as the number of BSI episodes demonstrating growth of a pathogen, divided by the total number of suspected BSI episodes. Multi-drug resistance (MDR) was defined as resistance to ≥ 3 antibiotics from different antibiotic classes, from predefined categories for *Enterobacterales*, *Staphylococcus aureus*, *Enterococcus* species, *Pseudomonas aeruginosa* and *Acinetobacter* species [26]. For *Salmonella* Typhi, MDR was defined as combined resistance to ampicillin, trimethoprim-sulfamethoxazole and chloramphenicol [27]. Decreased ciprofloxacin susceptibility (DCS) was defined as an MIC value for ciprofloxacin of > 0.06 µg/mL but < 1 µg/mL for *Salmonella* species [20]. A healthcare-associated infection (HAI) was defined as a confirmed bloodstream infection episode, sampled > 48 h after admission in the hospital [28]. Time-to-detection was defined as the time between incubation and first sign of growth, either visual signs in the BCB or growth on blind subculture. Time-to-colonies was defined as time between incubation and recovery of bacterial colonies on agar plate.

## Results

### Blood culture results

**Fig. 2:**
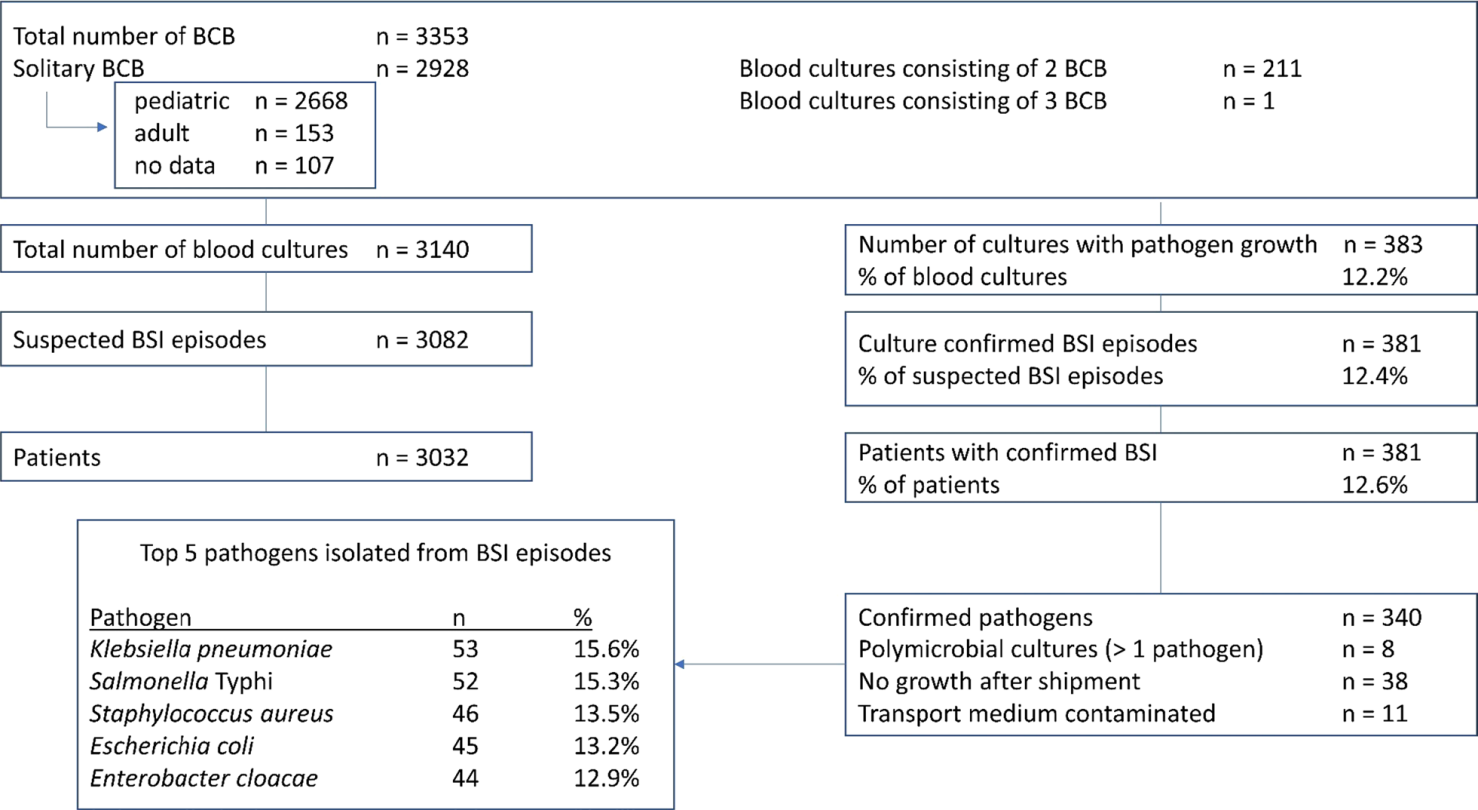
Breakdown of blood culture data from Boko, Benin. *BCB* blood culture bottle; *BSI* bloodstream infection

Between October 2017 and March 2020, a total of 3140 blood cultures were sampled from 3031 patients (Fig. 2).

Of these cultures, 11.7% (n = 367) were submitted by the CHUD in Parakou; the other cultures were sampled in Boko hospital (n = 2773). Median age of all patients was 2 years (interquartile range 0–6 years) (Table 1). Adults (> 15 years) were underrepresented and made up only 11.3% of the patient population (306/2705). Of patients whose sex was known (n = 2880), 44.7% was female (Table 1). A comparison of the patient population from Boko versus Parakou is presented in Additional file 1: File 5.

**Table 1:** Demographic and clinical data of study participants

Total number of patients	3032
Total number of suspected BSI episodes	3082
Number of suspected BSI episodes per hospital	
Boko hospital	2724 (88.4%)
CHUD Parakou	358 (11.6%)
Median age of patients	2
Patient sex	
Female	44.7% (1286/2880)
Male	55.3% (1594/2880)
Antibiotic treatment before culture	22.5% (684/3036)
Healthcare-associated infection	10.4% (318/3065)
Pathogen rate of suspected episodes	12.4% (381/3082)
Contamination rate	16.9% (565/3353)
Thick blood film positive (malaria diagnosis)	51.8% (1255/2423)
Thick blood film positive in confirmed BSI episodes	40.2% (101/251)
Presumed focus of infection	
Generalized	2088
Respiratory	7.9% (216/2730)
Purulent	2.4% (66/2730)
Central nervous system	1.8% (49/2730)
Abdominal	1.5% (40/2730)
Urinary/genital	1.3% (36/2730)

*CHUD* Centre Hospitalier Universitaire Departemental; *BSI* bloodstream infection

Out of 3082 suspected BSI episodes, 381 (12.4%) yielded one or more pathogens as identified in the laboratory of Boko hospital (Fig. 2). Of these, 340 were identified in Belgium with MALDI-TOF. Local identification in Boko was correct for 249 isolates (73.2%), albeit with 33 of these isolates being identified only up to genus level, and 26 isolates to a genus group level (e.g., non-fermenter). Identification up to group level occurred mainly in the beginning of the surveillance period (2017 and start of 2018). Correct identification up to species level was thus achieved for 190 isolates (55.9%). Among key pathogens, incorrect identification was most common for *Enterobacter cloacae* isolates (43.2% incorrect identification) and least common for *Salmonella* Typhi isolates (11.5% of isolates being incorrectly identified as other *Salmonella* serotypes (6/52) and only 1 isolate identified as other *Enterobacterales* species).

**Fig. 3:**
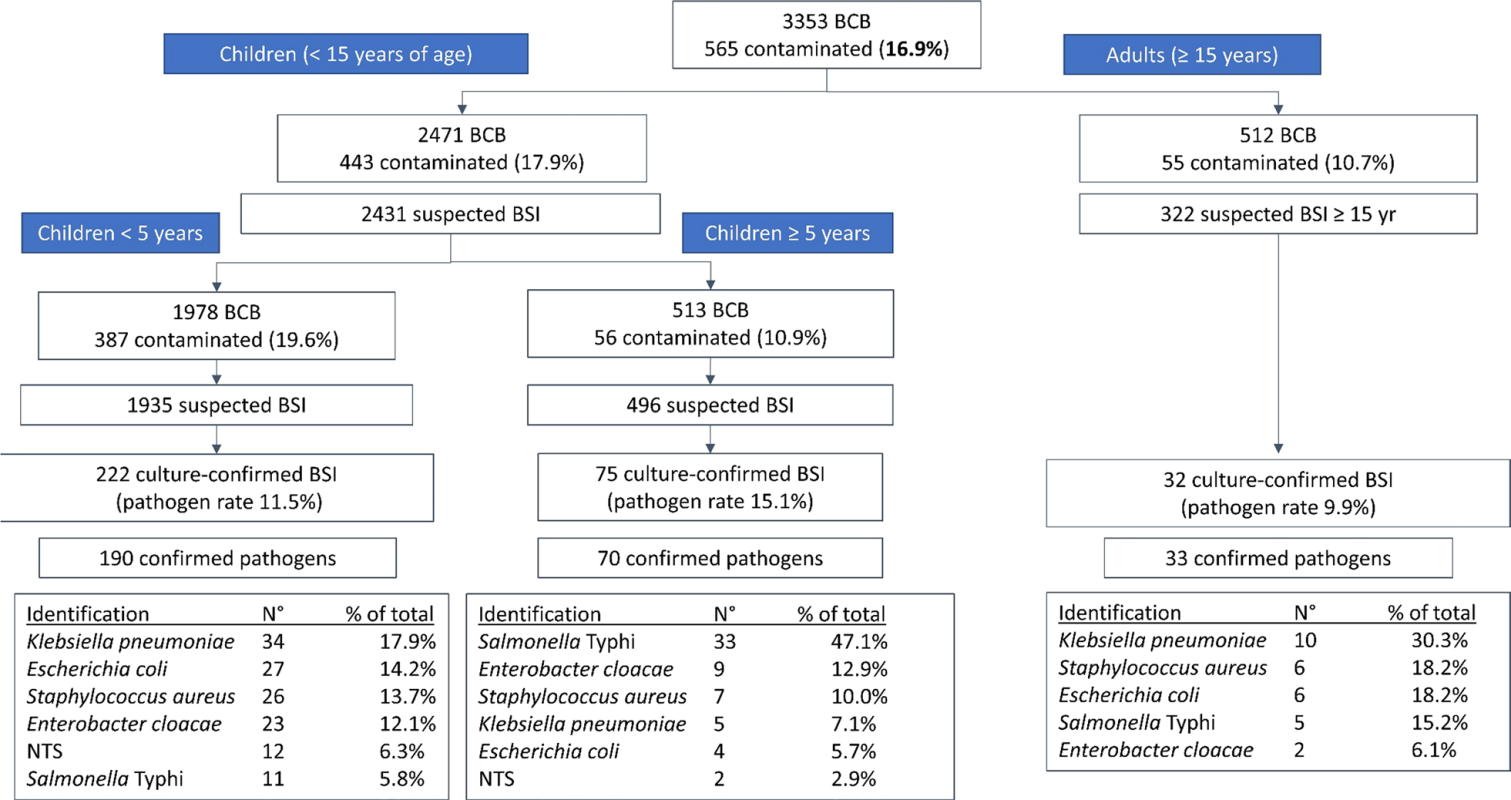
Detail of Fig. 2. Breakdown of blood culture data stratified by age of patient. *BCB* blood culture bottles; *BSI* bloodstream infection episode; *NTS* non-Typhi *Salmonella*. For more detail of children < 5 years of age, see Fig. 4

**Fig. 4:**
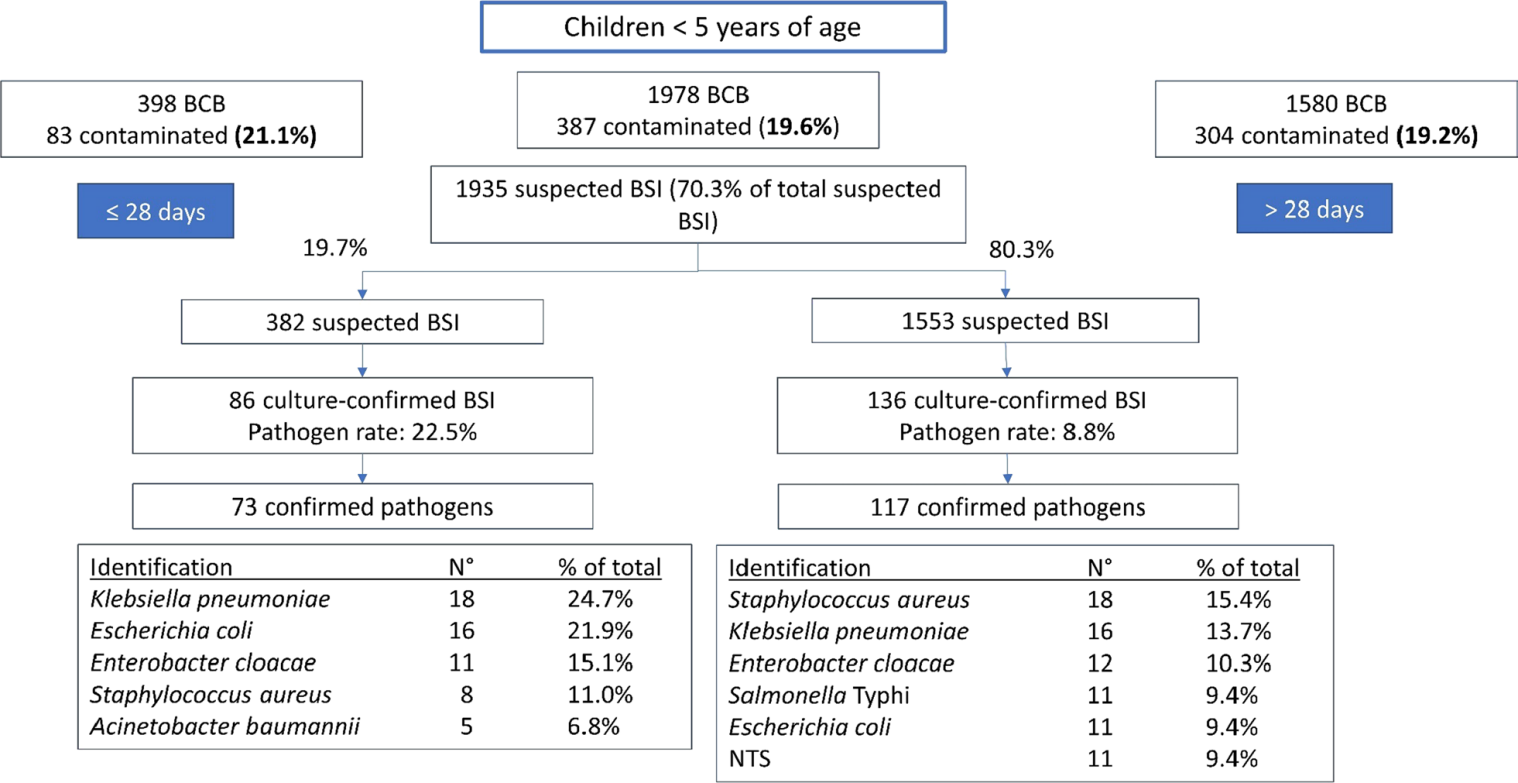
Further detail of Fig. 3; breakdown of data for children < 5 years age, stratified by age (≤ 28 days of > 28 days of age). *BCB* blood culture bottles; *BSI* bloodstream infection episode; *NTS* non-Typhi Salmonella

The most common pathogens were *Klebsiella pneumoniae* (15.6%), *Salmonella* Typhi (15.3%) and *Staphylococcus aureus* (13.5%). Key pathogens differed according to age; *Klebsiella pneumoniae* was the most common pathogen in neonates and adults, whereas *Salmonella* Typhi was the most important pathogen in children > 5 years of age (47.1% of pathogens) (Figs. 3, 4). Polymicrobial episodes were present in 2.1% of all grown BSI episodes (n = 8). HAI were more frequent in adults than children (42.4% of confirmed episodes versus 15.9%, p = 0.0005). All identified pathogens and their frequency can be found in the Additional materials (Additional file 1: File 6). The pathogens retrieved included some unexpected or rare pathogens, such as *Elizabethkingia anophelis* and *Burkholderia pseudomallei.* The latter pathogen was isolated from a 5-year old girl with fever but no localized symptoms according to the blood culture request form.

### Blood culture quality indicators

The overall pathogen rate was 12.4% (target 5–15%) [29], with higher pathogen rates among neonates and children between 5 and 15 years of age (Fig. 3). Contamination rate was very high (16.9%) and was substantially higher in children under 5 (19.2%) and neonates (21.1%) than in older children (10.9%) and adults (10.7%) (p < 0.001) (Fig. 4). The internationally recommended contamination rate is < 3% [29].

The volume of blood sampled per bottle was measured for 2248 BCB (67.0% of all bottles). Volumes sampled in different age categories (which have different volume requirements) are shown in Additional file 1: File 7 [4]. Underfilling was an important problem; it occurred for 64.9% of neonatal bottles (n = 185), 70.4% of bottles in children 1–36 months of age, 94.5% of bottles in children > 36 months of age, and 53.0% of adult bottles.

Median time-to-detection was 2 days (interquartile range 1–3 days). Implementation of a blind subculture on day 1 (March 2019) did not significantly improve time-to-detection or time-to-colonies: before blind subculture implementation, 75.5% (209/277) of growth detection and 48.4% (134/277) of colony growth was observed by day 2 of incubation, versus 63.9% (62/97) and 54.6% (53/97) respectively after the implementation of blind subculture.

Growth on blind subculture was more likely to be a contaminant (n = 138) than a pathogen (n = 67; out of 106 total pathogens). Nonetheless, contamination rate did not increase after implementation of blind subculture (contamination rate went from 18.0 to 15.3%).

### Clinical and laboratory variables associated with pathogen growth

Table 2 shows the association of different variables on the pathogen rate. Blood culture growth was associated with the hospital of sampling, even more so after adjusting for other variables (crude OR 1.90, CI 1.43–0.77; adjusted OR 2.04, CI 1.28–3.26). The laboratory confirmation of malaria (positive thick blood film) was inversely associated with odds of blood culture growth in Boko hospital (crude OR 0.67, CI 0.51–0.89; adjusted OR 0.64, CI 0.46–0.88). Neonatal cultures (crude OR 2.61, CI 1.70–4.03; adjusted OR 2.57, CI 1.51–4.39) and healthcare-associated infections (crude OR 1.92, CI 1.42–2.69; adjusted OR 1.52, CI 1.01–2.87) were also associated with growth. There was no significant association between presumed focus of infection (generalized, abdominal, respiratory, central nervous system, urinary or purulent infections, i.e., skin/soft tissue, osteomyelitis or abscesses) and odds of growth (Additional file 1: File 8).

**Table 2:** Association of different variables with odds of pathogen growth of blood culture, expressed as odds ratio [95% confidence interval (CI)]

Variable	Categories	Univariable analysis	Multivariable analysis
Age category	Adults	1	1
	Children 5–15 years	1.60* [1.03–2.48]	2.13** [1.30–3.50]
	Children < 5 years	0.85 [0.57–1.27]	1.14 [0.72–1.81]
	Neonates (≤ 28 days)	2.61*** [1.70–4.03]	2.57*** [1.51–4.39]
Sex	Female	1	1
	Male	1.01 [0.80–1.28]	1.12 [0.86–1.44]
Prior antibiotic treatment	No	1	1
	Yes	1.16 [0.90–1.49]	1.08 [0.79–1.48]
Healthcare-associated infection	No	1	1
	Yes	1.92*** [1.42–2.69]	1.52* [1.01–2.87]
Thick blood film (malaria diagnosis)^†^	Negative	1	–
	Positive	0.59** [0.45–0.77]	–
Hospital	Boko	1	1
	CHUD Parakou	1.90*** [1.43–2.53]	2.04** [1.28–3.26]
Blood volume cultured	Per extra mL^‡^	1.01 [0.98–1.04]	–

Statistical significance is indicated as follows: *p-value 0.01–0.05; **p-value 0.001–0.01; ***p-value < 0.001

*CHUD* Centre Hospitalier Universitaire Departemental

^†^Due to high amount of missing data from CHUD Parakou hospital, thick blood film results for this hospital were not included in multivariable analyses

^‡^The odds ratio expresses the relative increase/decrease in odds of pathogen growth per unit increase in blood volume (unit = 1 mL)

### Antimicrobial resistance

Antibiotic susceptibility results are presented only for bacterial species for which more than 30 isolates could be recovered. Tables 3 and 4 show susceptibility rates of Gram-negative and Gram-positive isolates, respectively. Resistance to third-generation cephalosporins was very high (> 70%) in both *Enterobacter cloacae* and *Klebsiella pneumoniae* isolates. Two *Escherichia coli* and two *Enterobacter cloacae* isolates showed resistance to meropenem by disk diffusion; MIC values determined with E-tests were 4 µg/mL and 8 µg/mL for the *Escherichia coli* isolates and 2 µg/mL and 4 µg/mL for the *Enterobacter cloacae* isolates. For all four carbapenem-resistant isolates, presence of a New Delhi metallo-beta-lactamase was demonstrated. Two of these isolates were recovered from patients from CHUD Parakou, a 60-year-old man and a 9-month-old boy. The other two isolates were recovered from neonatal boys in Boko Hospital. Two of these isolates (1 *Escherichia coli* and 1 *Enterobacter cloacae* isolate) showed resistance to all tested antibiotics.

**Table 3:** Antibiotic resistance rates (% of isolates intermediate or resistant to the antibiotic listed) of Gram-negative bacterial isolates (only species for which n > 30) and proportion of isolates showing specific antibiotic resistance patterns and mechanisms

Antibiotic	*Enterobacter cloacae* n = 44	*Escherichia coli* n = 49*	*Klebsiella pneumoniae* n = 58*	*Salmonella* Typhi n = 53*
Antibiotic susceptibility rates (% susceptible)				
Ampicillin	–	73.5%	–	30.2%
Amoxicillin-clavulanic acid	–	41.7%	81.0%	–
Piperacillin-tazobactam	16.3%	18.7%	25.9%	–
Cefuroxime	72.7%	40.8%	79.3%	–
Ceftriaxone	70.5%	12.8%	77.6%	0%
Temocillin	7.0%	11.0%	0%	–
Meropenem	4.5%	4.1%	0%	0%
Ciprofloxacin	75.0%	35.4%	70.7%	24.5%
Trimethoprim-sulfamethoxazole	76.7%	36.7%	15.5%	39.6%
Gentamicin	68.2%	24.5%	74.1%	0%
Amikacin	2.3%	0%	3.4%	–
Chloramphenicol	65.9%	24.5%	37.9%	39.6%
Azithromycin	–	–	–	0%
Antibiotic resistance mechanisms and patterns				
ESBL	68.2%	28.6%	79.3%	0%
Carbapenemase	4.5%	4.1%	0%	0%
Combined resistance carbapenems–fluoroquinolones	2.3%	2.0%	0%	0%
MDR**	79.5%	69.4%	87.9%	39.6%
XDR**	22.7%	8.2%	32.8%	0%
PDR**	2.3%	2.0%	0%	0%
DCS**	–	–	–	24.5%

*ESBL* extended spectrum beta-lactamase; *DCS* decreased ciprofloxacin susceptibility; *MDR* multidrug resistance; *XDR* extensive drug resistance; *PDR* pan-drug resistance

*Isolate numbers do not always correspond with pathogens retrieved from cultures as depicted in Fig. 2, because some cultures contained morphologically different isolates of the same species. These were counted as different isolates in the antibiotic susceptibility analysis when their antibiotic susceptibility patterns differed, but not in the overall analysis of key pathogens

**MDR, XDR and PDR are defined according to Magiorakos et al., except for *Salmonella* species [26]. For *Salmonella* species, MDR is defined as resistance against ampicillin, co-trimoxazole and chloramphenicol; XDR is defined as resistance to ampicillin, co-trimoxazole, chloramphenicol, third generation cephalosporins and azithromycin [27]. Decreased ciprofloxacin resistance is defined as MIC ciprofloxacin > 0.06 but < 0.5 [20]

**Table 4:** Antibiotic resistance rate (% of isolates intermediate or resistant to the antibiotic listed) of Gram-positive bacterial isolates (only species for which n > 30)

Antibiotic	*Staphylococcus aureus* n = 49
Antibiotic resistance rate (% intermediate or resistant)	
Penicillin	77.6%
Oxacillin	22.4%
Ciprofloxacin	16.3%
Clindamycin	0%
Doxycycline	20.4%
Erythromycine	2.0%
Linezolid	0%
Gentamicin	10.2%
Trimethoprim-sulfamethoxazole	16.3%
Vancomycin	0%
Antibiotic resistance patterns & mechanisms	
MDR	22.4%
MRSA	22.4%
Co-resistance oxacillin-ciprofloxacin	12.2%
Co-resistance oxacillin-gentamicin	10.2%
Co-resistance oxacillin-ciprofloxacin-gentamicin	10.2%
Co-resistance oxacillin-doxycycline	8.2%
Co-resistance oxacillin-ciprofloxacin-doxycycline	6.1%
Co-resistance oxacillin-trimethoprim-sulfamethoxazole	2.0%
Co-resistance oxacillin-erythromycin	2.0%

Other *Enterobacterales* tested (n = 6; *Proteus mirabilis*, *Pantoea dispersa* and *Klebsiella oxytoca*) showed uniform susceptibility to third-generation cephalosporins, ciprofloxacin and amoxicillin-clavulanic acid.

Antibiotic susceptibility rates of *Salmonella* Typhi are shown in Table 3. None of the *Salmonella* Typhi displayed resistance to third generation cephalosporins or azithromycin. Decreased ciprofloxacin susceptibility was present in 24.5% of *Salmonella* Typhi isolates (13/53). None of these isolates had MIC values for ciprofloxacin higher than 0.25 µg/mL. No MIC values higher than 4 µg/mL were found for azithromycin, which is well below the susceptibility cut-off of 16 µg/mL.

Methicillin-resistance *Staphylococcus aureus* (MRSA) made up 22.4% of *Staphylococcus aureus* isolates (11/49) (Table 4). All *Staphylococcus aureus* isolates were susceptible to clindamycin, linezolid and vancomycin (Table 4). Vancomycin MIC values ranged between 0.5 and 1.5 µg/mL. No inducible resistance to clindamycin was detected with the D-test.

Non-fermenters for which CLSI disk diffusion breakpoints were available, were also tested (13 *Acinetobacter* species, 15 *Burkholderia cepacia*, 6 *Pseudomonas aeruginosa*, 4 *Stenotrophomonas maltophilia*; data not shown). Of the *Acinetobacter* species isolates, 2/13 were resistant to meropenem; one of these isolates was resistant to all antibiotics tested. No carbapenemase enzymes were detected in *Acinetobacter* spp. *Burkholderia cepacia* isolates showed relatively low AMR rates, with only 1 isolate (6.7%) resistant to any of the tested antibiotics (co-resistance to ceftazidime, co-trimoxazole and minocycline). Of the *Pseudomonas aeruginosa* isolates, only 1 isolate showed increased resistance to beta-lactam antibiotics (intermediate susceptibility to piperacillin-tazobactam and ceftazidime).

### Clinical variables associated with AMR

Associations between clinical and demographic variables and AMR are shown in Table 5. Only prior antibiotic treatment was associated with occurrence of MDR (crude OR 2.23, CI 1.28–3.88; adjusted OR 3.33, CI 1.52–7.31). There was no association between the hospital of sampling and proportion of pathogens being MDR, nor was there an association between MDR and HAI.

**Table 5:** Association of different variables with odds of pathogens being multi-drug resistant (MDR), expressed as odds ratio [95% confidence interval (CI)]

Variable	Categories	Univariable analysis	Multivariable analysis
		Total	Total
Age category	Adults	1	1
	Children 5–15 years	0.56 (0.23–1.36)	0.47 (0.15–1.41)
	Children < 5 years	0.59 (0.26–1.36)	0.51 (0.18–1.45)
	Neonates (≤ 28 days)	0.85 (0.35–2.06)	1.10 (0.33–3.55)
Sex	Female	1	1
	Male	1.05 (0.64–1.73)	1.13 (0.32–2.03)
Antibiotic treatment < 24 h prior to sampling	No	1	1
	Yes	2.29** (1.32–3.98)	3.39 (1.54–7.44)
Healthcare-associated infection	No	1	1
	Yes	1.90 (0.94–3.83)	1.20 (0.41–3.54)
Hospital	Boko	1	1
	CHUD Parakou	0.58 (0.32–1.04)	0.42 (0.13–1.33)

*CHUD* Centre Hospitalier Universitaire Departemental

Statistical significance is stated as follows: *p-value 0.01–0.05; **p-value 0.001–0.01; ***p-value < 0.001

## Discussion

In this paper, we present blood culture and antibiotic resistance surveillance data from a secondary care hospital in Benin. The most commonly isolated bloodstream pathogen was *Klebsiella pneumoniae*. Among children 5–15 years of age, *Salmonella* Typhi was the most important bacterial pathogen, making up almost half of all isolates. This is in sharp contrast to other, similar settings in sub-Saharan Africa, where non-typhoid *Salmonella* are the main culprit of BSI in children, and much more common than *Salmonella* Typhi [27, 30–32]. HIV and malaria prevalence (both risk factors for non-typhoid *Salmonella*) are similar in Benin compared to other Western and Central African countries and can thus not explain this disparity [33, 34].

Some unexpected pathogens were recovered, such as *Burkholderia pseudomallei*, an important and lethal pathogen in South-East Asia but not known to be endemic in Africa. *Burkholderia pseudomallei* may indeed be more common in Africa than previously reported [35, 36]. It has been shown that many African countries provide the right ecological conditions for this environmental species to thrive, and Benin is among the countries where *Burkholderia pseudomallei* is predicted to be endemic but so far not reported [37]. Lack of widespread laboratory infrastructure in many African countries, especially in rural settings, may contribute to underdiagnosis of this pathogen.

On the other hand, no *Streptococcus pneumoniae* isolates were recovered in this study, although a systematic review from 2010 found it to be one of the most common bloodstream pathogens in Africa [38]. Other, related *Streptococcus* species were occasionally retrieved; therefore, the problem does not lie in lack of detection by the blood culture technique. This finding could be partially explained by vaccination; the pneumococcal conjugate vaccine has been implemented in Benin in 2011 and uptake has been good (> 80% 4 years after introduction) [39]. High antibiotic use prior to sampling is also a probable explanation, as these bacteria are usually highly susceptible to beta-lactam antibiotics and 22.5% of patients in this study had been administered antibiotics < 24 h prior to sampling.

Pathogen rates (shown in Fig. 3) were too high in children 5–15 years and neonates, indicating undersampling in these age groups [29]. Moreover, the stark difference between pathogen rates in Boko hospital and CHUD Parakou (11.3% versus 19.6%) demonstrate more appropriate sampling in Boko hospital, where these indications were explained and enforced during multiple visits.

Identification of pathogens was correct up to at least genus level for 65.0% of isolates identified by reference methods, and up to species/complex level for 55.9%. This actually compares favourably to other studies in LMIC, wherein conventional methods showed only 57.2% accuracy on genus level and 33.1% on species level for *Enterobacterales* [40]. As the present study also included pathogens difficult to identify up to species level with conventional methods (e.g., *Enterococcus* species, *Elizabethkingia anophelis*, *Acinetobacter* species), these results are encouraging. Moreover, identification up to group level occurred mainly at the start of the study, when identification procedures were not yet fully mastered. However, error rate was still quite high for some important pathogens, such as *Enterobacter cloacae*. These observations highlight the need for accurate, affordable and easy-to-use identification methods in this setting.

Underfilling was an important problem, especially in children, although volume was not significantly associated with blood culture growth (Table 2).

Contamination rates were dramatic and remained high despite repeated trainings, direct observation of sampling by the study team and change of the antiseptic. The contamination rates found are, however, in line with other reports from African countries, such as Malawi (19.6%), Ghana (13.2%) and the Gambia (23.4%) [41–43]. In Malawi, they were able to bring down contamination to 5% with implementation of a dedicated phlebotomy team [42]. Other LMIC hospitals have also been able to obtain very low contamination rates [25, 44].

Implementation of blind subculture did not lead to advantages in terms of time-to-detection or time-to-colonies. Given the extra workload associated with performing blind subculture and the increased risk of contamination, blind subculture was considered ineffective in this setting.

AMR rates in *Enterobacterales* were high, with more than half of isolates showing MDR. Rates of resistance to third-generation cephalosporins in particular were much higher than previously described for this region [45, 46]. Only 22.4% of *Klebsiella pneumoniae* isolates were still susceptible to ceftriaxone. The occurrence of NDM producing isolates in > 4% of *Enterobacter cloacae* and *Escherichia coli* isolates was in line with a meta-analysis on carbapenemase spread in Africa, which also found this carbapenemase to be relatively widespread in sub-Saharan Africa [47]. Carbapenem-resistant *Enterobacterales* and *Acinetobacter* are among the critical pathogens on the WHO priority pathogens list for research & development of new antibiotics [48].

*Salmonella* Typhi isolates were uniformly susceptible to ceftriaxone; MDR was found in nearly 40% of isolates and DCS was also an important problem affecting nearly a quarter of isolates. Rates of MRSA were along the lines or slightly lower than those in recent meta-analyses from LMIC (22.4% versus 30.6% and 29.5%, respectively) [45, 46].

AMR was associated with prior use of antibiotics, but not with HAI. This is surprising as the association between AMR and HAI is well-known and recognized [49]; the lack of association we found may be partly attributed to misclassification of some HAI as community-acquired. Neonatal infections occurring at day 0 or day 1 of life were classified as community-acquired according to our definition (see above); this definition may be flawed when babies are delivered in the hospital, when unclean delivery practices predisposes them to infection with hospital pathogens [50]. This may have led to underestimation of the association between HAI and AMR.

This study is among the first to report longitudinal, quality-assured blood culture data with antibiotic susceptibility results from a semi-rural setting in Western Africa, and the first of its kind in Benin. Due to the collaboration between Beninese and Belgian researchers, the majority of isolates could be tested in Belgium with reference techniques such as MALDI-TOF, which are often not available locally, not even at the tertiary level. Due to the large number of isolates retrieved, we can report reliable AMR rates for most important pathogens, such as *Salmonella* Typhi, *Escherichia coli*, *Klebsiella pneumoniae* and *Staphylococcus aureus*.

A limitation of this study is the lack of data on treatment and outcome of bloodstream infections in this population, which precludes any conclusions on the impact of AMR on patient outcomes. The World Health Organization (WHO) recommends follow-up on outcome data for surveillance of sepsis in LMICs [51]. In future blood culture surveillance studies, the inclusion of outcome data must be considered. In addition, there was substantial missingness of data for some clinical variables such as indication for blood culture sampling (11.3% missing; 349/3082) and blood film microscopy testing for malaria parasites (21.4% missing; 660/3082). Therefore, we chose not to include these variables in multivariable models to explain blood culture growth or AMR. Misclassification of contamination is possible; some coagulase-negative *Staphylococcus* species found could have been pathogens, especially in neonates [52]; on the other hand, some bacteria counted as pathogens may have been more often contaminant than pathogen in this setting; this has been described for more than 15% of *Enterococcus* species, *Streptococcus viridans* species and non-fermenters such as *Acinetobacter*, *Stenotrophomonas maltophilia* and *Pseudomonas* non-aeruginosa species [53]. Another potential bias is misclassification of healthcare-associated infections as community-acquired, as already discussed above.

Our findings may not be generalizable to very rural settings in Benin, given the close proximity to Parakou, the second largest city in Benin. Moreover, a substantial proportion of blood cultures were obtained from Parakou hospital. The strong preponderance of pediatric blood cultures also limits generalizability of our results to the overall adult population in Benin.

The high rates of AMR found in this study may have implications on empiric treatment of severe sepsis in this setting. Lack of availability of antibiotics such as carbapenems and vancomycin in LMIC effectively renders some of these infections untreatable [54]. This observation should be viewed in light of the concept of “difficult-to-treat” resistance; this is defined as resistance against all “first-line” antimicrobial agents, i.e., all beta-lactam (including carbapenems) and fluoroquinolone agents, necessitating the use of less effective or more toxic alternatives [55]. This concept is clinically more useful than MDR, as some MDR bacteria may still be susceptible to first-line agents, while other bacteria, due to intrinsic resistance, can be difficult to treat even when not formally classified as MDR. Especially in LMIC, “difficult-to-treat” resistance is relevant as LMIC settings often have very limited access to second-line agents such as colistin or amikacin and few means of monitoring plasma levels of toxic agents [56]. In the present study, only 1.0% of tested Gram-negative isolates (2 *Enterobacterales* and 2 *Acinetobacter baumannii* isolates) would be considered “difficult-to-treat” according to these definitions. This proportion is similar to reports from the United States [55]; however, the more general lack of access to antibiotics considered “first line” argues for a more broad definition of difficult-to-treat infections in LMIC compared to high-income countries.

Higher availability of broad-spectrum antibiotics, classified by WHO as “watch” or “reserve” antibiotics (AWARE classification), risks inducing more resistance to these antibiotics. However, in light of increasing AMR rates, the unavailability or unaffordability of these antibiotics in many African countries is detrimental to equitable access to healthcare. As the high antimicrobial resistance levels demonstrate, programs to increase access to antibiotics should go hand in hand with increased laboratory capacity for diagnosis of bacterial infections and antimicrobial stewardship programs in LMIC. The finding of unexpected pathogens highlights the need for widespread surveillance and accurate bacterial identification systems, adapted to LMIC settings. This study also demonstrated the feasibility of blood culture implementation in such settings, if adequately supported logistically and financially; the hospital was able to set the price for blood cultures at the same level as other low-cost tests (e.g., thick blood film) and this cost proved not to be a hinderance to sampling of blood cultures.

## Conclusion

Blood cultures were successfully implemented in a small hospital in a semi-rural setting in West-Africa, although contamination remained a problem. Blood culture surveillance revealed alarmingly high AMR rates, especially for Gram-negative bacteria. Unbiased estimates for AMR are difficult to obtain in LMIC, but our experiences highlight the potential for surveillance of AMR in the community through strengthening of clinical microbiology laboratories in remote, non-tertiary settings.

## Supplementary Information

**Additional file 1: File 1.** local laboratory work-up of blood cultures. **File 2.** reference isolate testing in Belgium. **Table S1.** Antibiotics tested for each of the pathogens, with disk diffusion (Neo-Sensitabs, Rosco Diagnostica) or E-test® (bioMérieux), as part of reference testing. **File 3.** Request and sampling form. **File 4.** Laboratory workup form. **File 5.** Comparison between patient population in Boko hospital and CHUD Parakou. **File 6.** All pathogens identified from October 2016 to March 2020, in alphabetical order, as confirmed by MALDI-TOF spectrometry. **File 7.** Blood volume sampled per bottle, shown in boxplots and stratified by age, as measured in 2248 of 3353 blood culture bottles (BCB). **File 8.** Association of presumed focus of infection (i.e., as recorded on the blood culture laboratory request form at the moment of sampling) with pathogen growth rate.

## Data Availability

The database for this manuscript will be made open access. Access requests for ITM research data can be made to ITM’s central point for research data access by means of submitting the completed Data Access Request Form. These requests will be reviewed for approval by ITM’s Data Access Committee (ITMresearchdataaccess@itg.be).

## Abbreviations

AMR: Antimicrobial resistance
AST: Antibiotic susceptibility testing
BCB: Blood culture bottles
BSI: Bloodstream infection
CHUD: Centre Hospitalier Universitaire Departemental
CLSI: Clinical & Laboratory Standards Institute
CNHU-HKM: Centre National Hospitalier Universitaire Hubert Koutougou Maga
DCS: Decreased ciprofloxacin susceptibility
HAI: Healthcare-associated infection
MALDI-TOF: Matrix-assisted laser desorption/ionization time-of-flight spectrometry
LMIC: Low- and middle-income countries
OR: Odds ratio
WHO: World Health Organization
XDR: Extended drug resistance
