# Supplementary material for: Blood culture surveillance in a secondary care hospital in Benin: epidemiology of bloodstream infection pathogens and antimicrobial resistance
Source: BMC Infect Dis. 2022 Feb 3;22:119. doi: 10.1186/s12879-022-07077-z (PMC8812239; doi:10.1186/s12879-022-07077-z)
Supplement: Supplementary file 1 — Additional file 1: File 1. local laboratory work-up of blood cultures. File 2. reference isolate testing in Belgium. Table S1. Antibiotics tested for each of the pathogens, with disk diffusion (Neo-Sensitabs, Rosco Diagnostica) or E-test® (bioMérieux), as part of reference testing. File 3. Request and sampling form. File 4. Laboratory workup form. File 5. Comparison between patient population in Boko hospital and CHUD Parakou. File 6. All pathogens identified from October 2016 to March 2020, in alphabetical order, as confirmed by MALDI-TOF spectrometry. File 7. Blood volume sampled per bottle, shown in boxplots and stratified by age, as measured in 2248 of 3353 blood culture bottles (BCB). File 8. Association of presumed focus of infection (i.e., as recorded on the blood culture laboratory request form at the moment of sampling) with pathogen growth rate. [file 12879_2022_7077_MOESM1_ESM.pdf]

Additional File 1: local laboratory work-up of blood cultures

Upon receipt in the laboratory, blood culture bottles (BCB) were incubated in a standard incubator (as opposed to a blood culture automate) and visually inspected twice daily for signs of growth, both in the broth (turbidity, hemolysis, gas formation) and by detecting colour change of the chromogenic CO_2 indicator at the bottom of the BCB. Upon signs of growth, a Gram stain was performed and a subculture on blood, chocolate and/or MacConkey agar was done, depending on result of the Gram stain. From September 2018 onwards, bottles were weighed upon reception at the laboratory with a Kern pocket balance (Kern & Sohn GmbH, Balingen, Germany) to determine sampled blood volume (see below). End of February 2019, a blind subculture (regardless of visual signs of growth) on day 1 of incubation (*i.e.* after one overnight incubation) of all BCB was implemented.

On-site identification and antibiotic susceptibility testing (AST) of bacteria was done using conventional phenotypic testing with use of Oxoid agar bases (ThermoFisher Scientific, Waltham, USA) and DiaTabs (Rosco Diagnostica, Taastrup, Denmark). Antibiotic susceptibility testing (AST) was performed by disk diffusion method, using Neo-Sensitabs (Rosco Diagnostica). Disk diffusion breakpoints by the Clinical & Laboratory Standards Institute (CLSI) were used and yearly updated to the latest version. When requested by the physician, a thick blood film (Giemsa stain) was assessed. Laboratory staff was well trained and participated to national quality assessment programs.

Additional File 2: reference isolate testing in Belgium

Upon arrival in Belgium, the isolates were identified using matrix-assisted laser desorption/ionization time-of-flight mass spectrometry (MALDI-TOF MS), using a Microflex™ device (Bruker Daltonics, Massasuchetts, USA) with MALDI Biotyper® software (MBT 7854 MSP Library) at the University Hospitals Leuven. *Salmonella* antisera of Pro-lab diagnostics (Richmond Hill, Canada) were used to serotype *Salmonella* isolates. *Salmonella* isolates that could not be serotyped with certainty were submitted for reference identification by the Belgian national reference laboratory for *Salmonella* (Scientific Service of Human Bacterial Diseases, Scientific Direction of Human Infectious Diseases, Sciensano, Brussels). Optochin disks (Rosco Diagnostica) were used to differentiate *Streptococcus pneumoniae* from other viridans *Streptococcus* species, after MALDI-TOF identification.

Antibiotics for which only Minimal Inhibitory Concentration (MIC) breakpoints were determined by CLSI were tested using E-tests® (bioMérieux). For azithromycin susceptibility testing in *Salmonella* Typhi, CLSI breakpoints were used for both disk diffusion (≥13 mm susceptible; ≤ 12 mm resistant) and MIC testing by E-tests (MIC ≤ 16 µg/mL susceptible). Vancomycin E-tests were performed for *Staphylococcus aureus* and when vancomycin disk was < 17 mm for *Enterococcus* species (to confirm vancomycin-resistant Enterococcus). Inducible resistance to clindamycin was tested for in *Staphylococcus aureus* isolates by performing D-test.

Quality control was performed each day of AST with the following American Type Culture Collection (ATCC) strains and strains obtained as part of external quality control programs from Sciensano: ATCC 25922 (*Escherichia coli*), ATCC 27853 (*Pseudomonas aeruginosa),* ATCC 25923 (*Staphylococcus aureus*; disks) and ATCC 29213 (*S. aureus*, E-test vancomycin). Carbapenemase and ESBL testing quality control was done with following reference strains: ATCC 700603 *Klebsiella pneumoniae* (ESBL), M/11720 *Klebsiella pneumoniae* (KPC), M/11721 *Klebsiella pneumoniae* (OXA48), ATCC BAA-2146 *Klebsiella pneumoniae* (MBL).

Additional files

Additional table 1: Antibiotics tested for each of the pathogens, with disk diffusion (Neo-Sensitabs, Rosco Diagnostica) or E-test® (bioMérieux), as part of reference testing

**Antibiotic & dose**	***Staphylococcus aureus***	***Enterococcus* species**	**Enterobacterales (except *Salmonella*)**	***Salmonella enterica***	***Pseudomonas aeruginosa***	***Acinetobacter* species**	***Burkholderia cepacia***	***Stenotrophomonas maltophilia***
Disk diffusion								
Penicillin 10 µg	x							
Ampicillin 10 µg		x	x	x				
Amoxicillin-clavulanate 20/10 µg			x					
Piperacillin-tazobactam 100/10 µg			x		x			
Temocillin 30 µg			x					
Cefoxitin 30 µg	x		x					
Cefuroxime 30 µg			x					
Ceftriaxone 30 µg			x	x		x		
Ceftazidime 30 µg			x	x	x	x	x	
Meropenem 10 µg			x		x	x		
Gentamicin 10 µg	x		x			x		
Amikacin 30 µg	x		x		x	x		
Ciprofloxacin 5 µg	x		x		x	x		
Pefloxacin 5 µg				x				
Chloramphenicol 30 µg			x	x				
Trimethoprim-sulfamethoxazole 1.25/23.75 µg	x		x	x		x	x	x
Clindamycin 2 µg	x							
Doxycycline 30 µg	x					x		
Minocycline 30 µg								x

Tetracycline 30 µg			x	x		x		
Erythromycin 15 µg	x							
Linezolid 30 µg	x	x						
Vancomycin 30 µg		x						
E-tests								
Azithromycin				x				
Chloramphenicol							x	
Ciprofloxacin				x				
Vancomycin	x							

Additional file 3: Blood culture request form used in Boko hospital

FICHE DE DEMANDE HEMOCULTURE

Veuillez utiliser une fiche par culture de sang

INSTITUTE OF TROPICAL MEDICINE ANTWERP

Données démographiques

Prénom : . . . . Nom : . . . .

Date de naissance: ___ ___ / ___ ___ / ___ ___ ___ ___ ou âge: . . . . (jours/mois/ans)
(DD/MM/YYYY)

N° d'hôpital : .... Sexe : M □ F □

N° de téléphone : .... Village : ....

Date d'admission: ___ ___ / ___ ___ / ___ ___ ___ ___ Référé: □ Oui □ Non
(DD/MM/YYYY)

Si référé, quelle facilité a référé : . . . .

Indications pour hémoculture:

1. □ Fièvre (axillaire T° ≥ 38°C) □ Hypothermie (axillaire T° ≤ 36°C)
2. Signes de gravité:
   - □ Hypotension □ Fréquence respiratoire augmentée □ Confusion
   - □ Suspicion d'une infection grave ou localisée:
     - □ Pneumonie □ Infections des voies urinaires compliquées
     - □ Méningite □ Abcès
     - □ Ostéomyélite □ Infection de la peau ou tissus mous
     - □ Infection abdominal
   - □ Suspicion d'une autre infection grave:
     - □ Le paludisme grave □ Autres: . . . .
     - □ Fièvre typhoïde
3. □ Infection néonatale

Début des symptômes: ___ ___ / ___ ___ / ___ ___ ___ ___ (DD/MM/YYYY)

Traitement:

Patient a pris des antibiotiques hier ou aujourd'hui : □ OUI □ NON

Type d'antibiotique (si connu): . . . .

Date de début de traitement antibiotique : . . . .

Patient a pris des anti-malariens: □ YES □ NO

Type de traitement anti-malarien (si connu): . . . .

Résultats TDR de paludisme :

Teste rapide de palu: □ Pas fait □ Négative □ Positive

Nom de médecin : . . . .

Tel : . . . . **Signature:** . . . .

Veuillez assurer que toutes les questions soient correctement remplies

Additional files

Additional file 4: Laboratory work-up form used in Boko hospital

FICHE LABORATOIRE POUR GESTION HEMOCULTURES

Veuillez utiliser une fiche par flacon

Nom de patient:
Numéro de bouteille hémoculture:

Date de réception: __ __ / __ __ / __ __ __ __ (DD/MM/YYYY)

Heure de réception: _ _ : _ _ (hh:mm)

Flacon d'hémoculture: ☐ Pédiatrique ☐ Adulte

Poids de flacon d'hémoculture : Avant prélèvement : __ __ , __ g; Après : __ __ , __ g

Inspection quotidienne du flacon (mettez un « X » au moment de virage):

Jour 1	Jour 2		Jour 3		Jour 4	Jour 5	Jour 6	Jour 7	Jour 8
	Matin	Après-midi	Matin	Après-midi					
Virage?									

Résultats de goutte épaisse:

Goutte-épaisse: ☐ Pas fait ☐ Négative ☐ Positive

Si positive, densité de parasites: . . . . /µl, *Plasmodium* species: . . . .

Gestion des hémocultures poussées:

Résultat coloration Gram: . . . .

Date: __ __ / __ __ / __ __ __ __ (DD/MM/YYYY)

Résultat Gram communiqué au clinicien: ☐ Oui ☐ Non

Communiqué par (initiales) : . . . . à ....

Résultats de cultures sur gélose et résultats identification:

Identification définitive: . . . .

Effectué par (initiales) : . . . . Date : __ __ / __ __ / __ __ __ __ (DD/MM/YYYY)

Résultats identification téléphoné au clinicien : ☐ Oui ☐ Non

Additional File 5: Comparison between patient population in Boko hospital and CHUD Parakou

	**Boko hospital**	**CHUD Parakou**	**p-value for difference**
**Total number of patients**	2676 (88.3% of total)	356 (11.7% of total)	-
**Total number of suspected BSI episodes**	2724 (88.4% of total)	358 (11.6% of total)	-
**Median age of patients**	2	3	p < 0.001
**Percentage female**	44.9% (1167/2599)	42.3% (119/281)	p = 0.39
**Antibiotic treatment before culture**	20.5% (550/2683)	37.9% (134/354)	p < 0.001
**Healthcare-associated infection**	9.2% (250/2717)	19.0% (68/358)	p < 0.001
**Pathogen rate of suspected episodes**	11.4% (311/2724)	19.6% (70/358)	p < 0.001
**Contamination rate**	17.0% (499/2943)	16.1% (66/410)	p = 0.72
**Thick blood film positive (malaria diagnosis)**	54.8% (1244/2270)	7.2% (11/418)	p < 0.001
**Thick blood film positive in confirmed BSI episodes**	45.9% (100/218)	3.0% (1/33)	P < 0.001

CHUD = Centre Hospitalier Universitaire Departemental. BSI = bloodstream infection

Additional File 6 : all pathogens identified from October 2016 – March 2020, in alphabetical order, as confirmed by MALDI-TOF spectrometry.

**Species**	**Number of isolates**
Achromobacter species	1
Achromobacter xylosoxidans	1
Acinetobacter baumannii	10
Acinetobacter species	3
Aerococcus viridans	5
Brevundimonas diminuta	1
Burkholderia cepacia	14
Burkholderia pseudomallei	1
Candida lusitaniae	1
Candida parapsilosis	1
Candida tropicalis	1
Elizabethkingia anophelis	1
Enterobacter cloacae	44
Enterococcus casseliflavus	2
Enterococcus faecalis	4
Enterococcus faecium	8
Enterococcus gallinarum	1
Escherichia coli	45
Klebsiella oxytoca	2
Klebsiella pneumoniae	53
Lactococcus lactis	2
Leclercia adecarboxylata	1
Moraxella osloensis	1
Moraxella species	1
Ochrobactrum anthropi	1
Ochrobactrum species	1
Pantoea dispersa	1
Proteus mirabilis	2
Pseudomonas aeruginosa	5
Pseudomonas stutzeri	1
Salmonella Enteritidis	8
Salmonella Herston	1
Salmonella Typhi	52
Salmonella Typhimurium	5
Staphylococcus aureus	46
Stenotrophomonas maltophilia	2
Streptococcus agalactiae	1
Streptococcus gallolyticus	1
Streptococcus mitis/oralis	2
Streptococcus parasanguinis	2
Streptococcus pyogenes	1
Weissella confusa	2

Additional File 7: **blood volume sampled**

Blood volume sampled per bottle, shown in boxplots and stratified by age, as measured in 2248 of 3353 blood culture bottles (BCB). Volume calculation was based on difference between filled bottle weight and bottle weight before filling. The solid line in the box represents the median volume, the "X" indicates the mean volume. Volume is indicated on the Y-axis in mL.

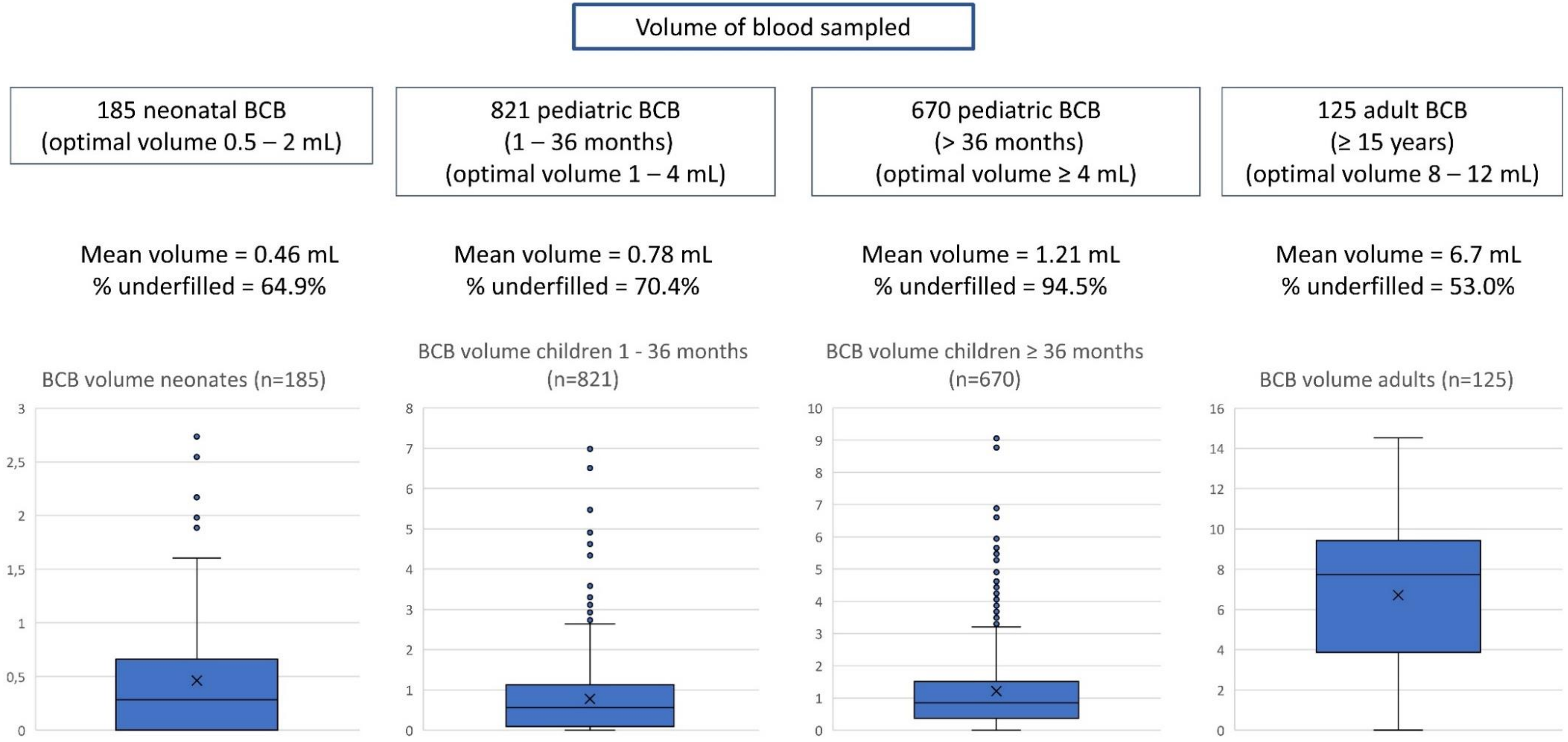

Abbreviations: BCB = blood culture bottle

Additional File 8: Association of presumed focus of infection (*i.e.,* as recorded on the blood culture laboratory request form at the moment of sampling) with pathogen growth rate. Generalized infection: no localized infection indicated. The p-value reflects the statistical significance of the difference in pathogen rate between generalized infections (no focus) and the possible foci of infection.

**Blood culture result**	**Presumed focus of infection**					
	Generalized*	Abdominal	CNS	Purulent**	Respiratory	Urinary/genital
Growth of pathogen	284 (12.2%)	3 (7.5%)	6 (12.2%)	12 (18.2%)	27 (12.5%)	6 (16.7%)
No growth or growth of contaminant	2049	37	43	54	189	30
Total	2333	40	49	66	216	36
p-value	-	0.367	0.998	0.154	0.918	0.427

* No localized infection indicated on request form
** Suspicion of skin/soft tissue infection, osteomyelitis or abscess as indicated on the request form
Abbreviations: CNS = central nervous system.
